# Where in the value chain are we losing the most food? The case of wheat in Jordan

**DOI:** 10.1007/s12571-019-00962-7

**Published:** 2019-08-16

**Authors:** Basel F. Y. Khader, Yigezu A. Yigezu, Mahmud A. Duwayri, Abdul Aziz Niane, Kamil Shideed

**Affiliations:** 1 Department of Agriculture, Scientific Research Support Fund, Jordanian Ministry of Higher Education, P.O Box 2680, Amman, Jordan; 2 International Center for Agricultural Research in the Dry Areas (ICARDA), PO Box 950764, Amman, Jordan; 3 University of Jordan, P.O Box 11942, Amman, Jordan; 4 International Center for Agricultural Research in the Dry Areas (ICARDA), Beirut, Lebanon

**Keywords:** Pre-harvest loss, Postharvest loss, Food waste, Bread, Wheat, Value chain

## Abstract

Efforts to increase global food supply through increased productivity and intensity of cropping are well documented. However, the literature on measurement of food losses and wastage and techniques to reduce them is scanty. This study aimed at providing credible evidence on the levels of food losses and wastage at each node along the entire wheat value chain in Jordan - from farm to fork. The “life cycle of food” approach, along with standard protocols developed in line with international initiatives led by the World Resources Institute (WRI) were used for physical measurements and estimation of losses at each node. Our results show that 34% of the total wheat supply in Jordan (both from local production and imports) is lost or wasted – costing the country about US$105 million per year, which is also associated with high levels of losses in natural resources. We found that postharvest losses are more important in Jordan where, at a level of 12.95%, wastage during consumption by households ranks first. Households reported that 67% of the household food waste was fed to animals. This means Jordan is losing 43% and 48% respectively of total protein and energy for every 1US$ spent on bread that is fed to animals instead of barley. These results call for a concerted effort by individuals, civic societies, NGOs and the government towards awareness raising and measures targeting reduction of wastage, especially during consumption. The Government of Jordan has recently reviewed the subsidy on bread, raising hopes that it will reduce consumption losses.

## Introduction

Food security is one of the major challenges facing humanity today. According to the definition provided by the world food summit of 1996, food security exists “when all people at all times have access to sufficient safe nutritious food to maintain a healthy and active life” (WHO 2014). Rapid population growth in the face of a shrinking productive resource base, including cultivatable land and irrigation water, makes the challenge increasingly difficult. World population is expected to reach 9.7 billion by 2050 (UN-DES 2017), which means there will be 33% more human mouths to feed, most of which will be in the poorest countries of the world (FA 2013). In order to meet the projected food demand in 2050, food supplies would need to increase by 60%.

Food availability can be increased using one or a combination of the following approaches: i) increased production through area expansion; ii) increasing productivity per unit area through intensification (defined here as increased amounts of inputs per unit area); and iii) reduction of food loss and wastage from the field to the fork; (iv) increasing cropping intensity by using the same land to produce more than one crop a year. However, there is generally limited scope for expansion of arable lands and when it is possible, it comes at the expense of environmental health (Pardey 2011). Increasing productivity through intensification is therefore the major route that had been taken to address problems related to food security and considerable achievements have been recorded over the years. However, the potential of genetic improvements and improved agronomic practices in increasing productivity seems to be approaching the right tail of the sigmoid curve for agricultural productivity. For instance, the global average annual productivity growths in the three major crops (rice, maize, and wheat) have been much lower for the period 1990–2007 than that for the period 1960–1990 (Pardey 2011).

If the additional wheat demand by 2050 is to be met, wheat yields have to grow by 1.4% per year. This means breeders will need to increase average global wheat yield by 0.7% per year - a rate of progress that most breeding programs are struggling to achieve, and agronomists will have to achieve yield gains of another 0.7% (WHEAT 2016). Achieving these targets is even more challenging in the dry areas (including Jordan–which is the subject of this study) where agricultural production is highly constrained by water scarcity, moisture stress and drought, and land limitation and degradation. Therefore, a multifaceted approach involving the exploration and exploitation of the potential with other alternative options that will complement the additional food supply from genetic gains is essential if the goal of making adequate food available for the growing world population is ever to be met.

Reducing food losses and wastage is one such option, the potential of which needs to be explored and fully exploited. Based on estimates by the Food and Agriculture Organization of the United Nations (FAO), about 1.3 billion tons of food is globally wasted or lost per year (Gustavsson et al. 2011) with a total value of roughly $250–$440 per year per household (Wilson and Wilson 2009). Reduction of these losses would lead to a substantial increase in the amount of food available for human consumption and an increase in global food security (WB 2008; Trostle 2010). To achieve the goals of food security, we must reduce food losses throughout the supply chain for each crop. About 1.2–2 billion metric tonnes (30–50% of global production) is estimated to be lost every year which has the potential to provide 60–100% more food for consumption. This implies wastage of 550 billion m^3^ of water and 1% to 1.5% of global energy (Fox and Fimeche 2013).

Around 25 million tons of wheat is estimated to be lost post-harvest, out of which, 46% is believed to occur in developing countries (Baloch 1999). Generally, developing countries suffer higher levels of food losses (both in quantity and quality) than developed countries - mainly due to widespread use of traditional methods of harvesting, handling, processing and storage of agricultural products.

Food waste remains a relatively unexplored topic especially in the Middle East in general and in Jordan in particular (Obeidate et al. 2015). While there are rough estimates of the total global food losses (Fox and Fimeche 2013), most of them are either based on expert opinions and hence vary widely or they do not provide clear explanation on what was being estimated, how the estimation was made, and when (Kitinoja and Kader 2015). More importantly, only a few of them provide detailed breakdown in terms of where in the value chains exactly these losses are taking place. Food losses and waste can happen at different nodes of the value chain, namely: in the field during the growing period of the crop, at harvest (due to timing, method and efficiency related problems), at storage, transport, processing, marketing, and consumption. Having estimates of losses at each of these nodes will be highly valuable for policy making and targeting and prioritizing interventions.

Some studies (see for example, Jones et al. 2018; Goldsmith et al. 2015; Sattar et al. 2015; Baloch et al. 1994; Sawhney 1988) have followed scientifically defendable protocols, approaches and established theories for measuring and analyzing losses. However, they are limited to only certain nodes or specific aspects of losses in a given node along the value chain, failing to provide the whole picture in one or more value chains. Recently, a number of studies that synthesize existing information from past studies on postharvest losses for horticultural crops (including meta-analyses) have characterized food losses (Gustavsson et al. 2011; Lipiniski 2013; Affognon et al. 2015). The resulting estimates are widely disparate, and the UN FAO SAVE FOOD Initiative currently uses the figures of 45% for losses of both roots/tuber crops and fruits/vegetables. Many international development authorities (e.g. the UNFAO, the World Bank and USAID) and journal articles citing primary works typically quote a general range of 30 to 50% postharvest losses. However, the sources of these estimates are not known (Kitinoja and Kader 2015).

Food losses are usually the unintended results of technical and management limitations in agricultural production processes, infrastructure, storage, transport, packaging, and/or marketing while food waste refers to food that is of good quality and fit for consumption but does not get consumed because it is discarded―either before or after it is left to spoil (Lipiniski 2013). Food waste typically, but not exclusively, takes place at the retail and consumption stages in the food value chain. It is usually the result of negligence or a conscious decision to throw food away. In terms of their nature, losses and wastes can be broadly classified into two: quantitative and qualitative. Losses can also be classified in terms of their utilization/disposal which includes the yield gap between what farmers are actually producing/harvesting and what was possible if they were to use optimal management practices; total loss (food going to landfills); diverted use (food used as feed, or for compost or biogas production and hence is no more available for direct human consumption); or food utilized sub-optimally (without capturing its full consumption and/or nutritional value(s)). Losses depend on several factors such as environmental conditions, method and duration of storage, methods of processing and the inherent features of the different nodes along the value chain in a given environment.

This paper attempts to quantify and provide credible evidence on the levels of food losses and wastage at each node along the entire wheat value chain - from farm to fork in Jordan. By so doing, the paper helps to prioritize investment in research into loss reduction interventions including the development and utilization of appropriate and advanced techniques, policy and institutional changes and awareness raising among citizens. This study should not be considered as a value chain analysis, but simply a study that attempts to measure food loss or wastage at each node of the wheat value chain. For the purpose of our study, we define food loss and waste as wheat-based food that becomes unavailable for direct human consumption in Jordan after it is imported or during and after local production. While we are aware of the ongoing debate about whether food put to alternative uses (such as animal feed) is a waste or not (see for example Bellemare et al. 2017), there does not seem to be a consensus. Therefore, in this paper, we have decided to measure it and present it as waste and let the reader decide on whether to include it in the total loss or not. Moreover, we provide detailed comparison on the opportunity cost of using processed wheat (particularly bread) for feed as opposed to barley, which is the main feed item in Jordan and show the implications in efficiency in terms of calorie and protein intakes of the animals.

Given the limited time and resources that were available, this study does not include all types of possible losses/wastes. Some of the losses/wastes may not even be relevant to the study area. For example, sprouting and weathering are more relevant in high rainfall and humid areas than in the dry areas covered in our study. Specifically, this study is limited to the measurement of quantitative food losses and wastes at the different nodes of the wheat value chain in Jordan including losses that were not realized due to technical and management-related shortcomings as well as food inadvertently or negligently wasted during processing, marketing and consumption.

Protocols developed in line with international initiatives (Hanson et al. 2016) were used for physical measurements and estimation of losses during pre-harvest, harvest, storage, transport, and consumption at restaurants. Surveys and company records were used to collect data that were used for estimation of losses during processing, marketing, and consumption at households. Micro-level estimates were aggregated to national levels using area and population weights. The findings of this study are expected to be useful to policy makers, donors, researchers and community-based organizations that work in the areas of food security and nutrition. The results are also expected to stir useful discussions nationally, regionally and globally, in terms of raising awareness as well as priority setting for research investment.

## The wheat sector in Jordan

Jordan is generally a net food importer, despite self-sufficiency in a few food items such as olives, olive oil, tomatoes, goat meat, fresh milk and eggs. The biggest gap between production and consumption is cereals, as Jordan produces only 3% and 3.6% of its total food needs in wheat and barley respectively (Badran et al. 2018). Price support policies for both inputs and outputs play a vital role in shaping the production system in the rainfed region of Jordan. The government price support system for encouraging production of field crops (mainly wheat and barley) has triggered a huge production process in areas that are not suitable for field crop production. On the one hand, this process has destroyed a major part of the fragile vegetative cover in the rangelands, and on the other, it has increased the livestock numbers, predominantly those of small ruminants. High supplementary feed costs encouraged by the government barley subsidies and falling forage availability are major constraints both on the profit margins of producers and in the competitiveness of their products at national and international markets. Twenty years of subsidy and ease of transport around the desert have encouraged the livestock industry to become dependent on barley, which accounted for 63% of feed costs for producers. Barley remains cheaper than any other alternative cereal such as maize (Sidahmed et al. 2012). However, a substantial amount of bread is also being used as animal feed (Khraishy and Beillard 2018).

Agricultural production, local or international transport, processing, storage and distribution is very sensitive to energy costs. Energy costs make up 15% to 22% of total food production costs in Jordan. Though it is still important to the country, the impact of energy costs on the wheat value chain in Jordan is believed to be relatively small because Jordan depends mainly on wheat imports (Badran et al. 2018). In 1996, the government implemented an economic adjustment program promoted by the IMF, which focused mainly on subsidy expenditures. Bread subsidies were particularly targeted since international wheat prices surged from $175 to $280 per ton in 1995. Within a few days, the price of bread and fodder more than doubled and consequently, the government introduced a compensation scheme to protect the poor. Unfortunately, the compensation was far from sufficient, and it was actually offset by price increases for dairy products. This was because fodder was a major input in dairy production (JIEW 2014). The general food subsidies were believed to be only marginally beneficial to the poor. After 1992, there was a shift to progressive cash transfers, which mainly targeted the unemployed and those not physically able to take up available jobs. The shift is said to have led to better consumption outcomes because of better targeting. This program would, a-priori, have had a greater impact on poverty reduction than general food subsidies because targeting of the poor was quite successful (Seijaparova and Pellekaan 2004). However, by 2002, several other factors, including inflation, caused a significant portion of the non-poor population to get very close to the poverty line (JIEW 2014). To make it worse, the pressure from IMF to eliminate fuel subsidies caused the government to take an initiative to reduce them gradually over the period 2005–2008, which was followed by another round in 2012 (Badran et al. 2018).

Historically, bread subsides in Jordan were implemented through the subsidization of flour. For example, in 2017, the market price of wheat flour was about $332 per metric ton (MT) while the size of the subsidy was approximately $71/MT. Large variation in flour prices has reportedly fomented a black market and illegal practices including tax evasion (Khraishy and Beillard 2018). In 2017 Jordan allocated approximately $170 million to a bread subsidy program. As of January 2018, the Jordanian government moved away from subsidizing the flour into cash transfers to targeted families and increased the price of subsidized bread. Therefore, large bakeries welcomed the Jordanian government’s measure to lift flour subsidies because the reform is expected to help put an end to subsidized wheat flour leakages that distort the market. When the new policy is fully implemented, bakeries that traded/resold subsidized wheat flour are expected to drop out of the market (Khraishy and Beillard 2018).

### Description of the wheat value chain in Jordan

With a 5-year average yield of 1.23 MT/ha, total annual domestic production stands at 23,420 MT - enough to cover only a few weeks of national demand. Total wheat imports during the same period amounted to 794,000 MT. In the face of a growing population, water shortage, and declining land productivity, the share of domestic production in total national wheat supply in Jordan is getting smaller with time - making the country increasingly import dependent (Table 1).

**Table 1:** Wheat area, local production and wheat imports wheat (2014)

Year	Total area planted to wheat (000′ ha)	Total harvested wheat area (‘000 ha)	Average Yield from harvested area (tonne/ha)	Total wheat production (‘000 tonnes)	Total wheat imports (‘000 tonnes)	Share of local production in total wheat supply (%)
1993	67.92	37.57	1.52	57.09	667.10	9%
1994	42.45	29.71	1.58	46.85	508.10	9%
1995	51.23	40.56	1.44	58.45	335.40	17%
1996	32.93	28.35	1.51	42.67	584.00	7%
1997	56.89	37.92	1.10	41.78	587.10	7%
1998	50.46	28.83	1.25	35.97	857.20	4%
1999	50.66	4.09	2.26	9.25	393.50	2%
2000	47.64	18.20	1.40	25.43	584.10	4%
2001	44.36	13.45	1.43	19.29	583.00	3%
2002	42.70	32.75	1.34	43.77	618.40	7%
2003	50.06	29.78	1.43	42.52	720.00	6%
2004	34.53	10.73	1.23	13.16	670.40	2%
2005	38.58	29.29	1.17	34.36	712.90	5%
2006	41.86	26.92	0.85	22.92	591.20	4%
2007	20.76	20.76	1.01	20.99	1,011.10	2%
2008	24.80	12.46	0.63	7.83	1,065.80	1%
2009	24.05	15.88	0.79	12.48	624.70	2%
2010	30.01	21.47	1.03	22.12	489.60	5%
2011	19.30	14.33	1.38	19.80	1,076.60	2%
2012	21.30	15.50	1.24	19.20	906.30	2%
2013	26.24	21.38	1.33	28.51	833.40	3%
2014	26.68	23.02	1.19	27.45	949.67	3%
10-Year-Average	27.36	20.10	1.06	21.57	729.68	3%
5-Year-Average	24.70	19.14	1.23	23.42	794.74	3%

Source: Department of Statistics, Agricultural Report, Amman-Jordan, 2014

With the few exceptions of those who store some wheat grain in their own facilities, most domestic farmers transport their produce to aggregation centers administered by agricultural co-operative societies (ACS). Then, the ACS sell part of the grain to the Ministry of Industry and Trade (MIT) for consumption and partly to the Ministry of Agriculture for use as seed in the next season. In 2014/15, the price of grain sold for consumption was 370 Jordanian Dinars (JD) per tonne while that of grain sold as seed was about 420 JD/tonne (1 JD = 1.41 US Dollars). In the most recent (2015/2016) season, only 11,863 tonnes of locally produced wheat was delivered to two public silos.

Wheat imported into the country is first stored in the storage facility at the port city of Aqaba where there are 150 silos with a total capacity of 140 thousand tonnes. It is then transported by land to three inland public storage facilities with a total storage capacity of 465 thousand tonnes, namely: 1) Irbid where there are 150 silos with a total capacity of 140 thousand tonnes; 2) Russeifa which has total capacity of 130 thousand tonnes and which is divided into four concrete silos of 9.4-thousand-tonne capacity each and ten metal silos of 9.3-thousand-tonne capacity each and; 3) Jowaydeh which contains 150 silos with a total capacity of 140 thousand tonnes. The average size of a typical silo in the country is 1300 tonnes. During the storage period, the minimum, average and maximum monthly ambient temperatures are usually 15 °C, 32 °C and 44 °C, respectively.

As locally produced wheat does not usually meet the minimum quality specifications, the silos always mix it with the imported wheat at a 10% to 90% ratio (the imported wheat constituting 90% of the mix) and store the mix as a single bulk with no quality differentiation. Usually, grain is stored in the silos for between 1 and 3 months with an average duration of 2 months. Out of the total wheat supply in the country, 82% is milled into flour. When wheat grain leaves the storage facilities, it is transported directly to grain mills. Jordan has a total of 16 wheat grain mills, 15 of which belong to the private sector while one (called Jwaydeh Mill) is public. The combined capacity of all 16 mills is about 120 tonnes/h.

**Fig. 1:**
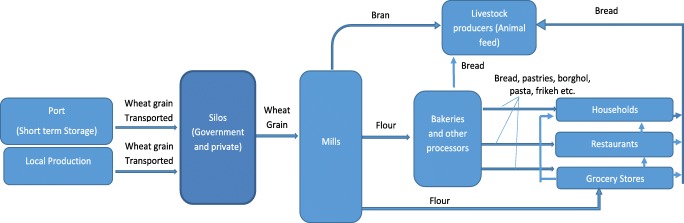
Description of the wheat value chain in Jordan. Source: depicted by authors

There are around 2100 bakeries (all private) which get their flour supplies from the mills to produce many kinds of bread. Most (96%) of the flour is used for the production of local bread called *Khubz* of different types where 75% (with high bran content and considered inferior in quality) is used to produce the common bread (locally called *mowahad Khubz*) which is sold at a highly subsidized price of 0.16 JD kg^1^; 15% (with slightly lower bran content) is used for the production of another type of common bread (locally called *mohassan khubz*), which is of a slightly higher quality and is sold at a price of 0.25JD/kg; 5% (first grade flour with very low bran) is used for the production of even higher qualities of bread (locally called *sharkh* and *hamamand Khubz*), which are sold at prices of 0.7JD/kg and 1JD/kg respectively. Four percent of the products of mills are used to produce pasta and semolina which are distributed in super markets. The remaining 1% is used to produce a wide variety of pastries, which are sold at a wide range of prices (1JD to 20 JD) depending on their kind and quality. Bakeries and pastry houses sell their products to households, grocery stores, and restaurants. The prices of wheat products are controlled by the Ministry of Industry and Trade, and there are penalties for the discordant bakeries/retailers that are not adhering to the prices set by the Ministry. Figure 1 depicts the wheat value chain in Jordan.

### Food loss and wastage in the wheat value chain

Pre and post-harvest food loss is defined as measurable qualitative and quantitative food loss along the value chain, starting at the time of harvest up to its consumption or other uses (Hodges et al. 2011). Food losses can be quantitative as measured by decreased weight or volume or can be qualitative such as reduced nutrient value and unwanted changes in taste, color, or cosmetic features of food (Buzby and Hyman 2012).

AOAD (2013) is one of the few papers that provides estimates of losses in the Arab world for different food groups (cereals, legumes, vegetables, etc.) from production to consumption. The document reported that 30% of total cereal production is lost between production and consumption. While the report provides estimates of loss at the different nodes of the value chains, it does not provide estimates for specific commodities (e.g. wheat, barley, tomatoes, etc.). More importantly, the document does not provide adequate description of how the estimates were generated.

Food losses along the wheat value chain covering harvest and postharvest operations, the latter consisting of transport, storage, processing, to consumption are the focus of this study. To the best knowledge of the authors, studies by Obeidate et al. (2015), Al Rawi (1989) and Snober et al. (1985) are the only ones conducted in Jordan on this subject for field crops. Losses differ across crops and locations. In a study of cereals, Al Rawi (1989) found that 1% loss occurred due to shattering, bird attack and other factors before harvest while grain losses due to mechanical harvesting were 7.87%. Grain losses due to storage in silos were 0.66% for wheat (after three months) and 2.37% for corn (after six months). Our calculations using data from a study of 1644 Jordanian consumers (Obeidate et al. 2015) showed very high levels (28.45%) of bread waste occurred during consumption.

The objective of this research was to measure the total wheat loss that occurs at each node across the entire wheat value chain in Jordan from the farm (for local production) and port (for imports) to the fork. Losses start to occur in the field before grain is harvested. These losses are hereafter classified into two groups, namely: management losses (the first node) and preharvest losses (the second node). Management losses take the form of yield gaps during crop growth that arise from farmers’ deviation from optimal management practices including planting date, seeding rate, the method of planting (broadcasting, row planting or seed drilling), types, amounts and timing of fertilizers, pesticide and herbicide use, and types of varieties used as well as losses occurring due to farmers’ skill gaps. The crop losses during the growing period that result from damage by diseases, weeds and insects are also part of management loss. Preharvest losses represent losses incurred after the crop has completed its growth period (i.e., between the period of physiological maturity of the crop and actual harvest date). These losses occur due to natural, varietal and management related causes, including shattering and insect and bird attack. Preharvest losses can be easily prevented if the farmers harvest at physiological maturity.

Losses during storage occur due to a number of factors that characterize the biophysical environment in the storage unit including temperature, humidity, insect or mold activity, birds and rodents, and grain respiration. The effects of each of these possible sources of loss depend on the duration and specific month and season (winter, spring, summer or fall) of the storage period and the genotype.

**Fig. 2:**
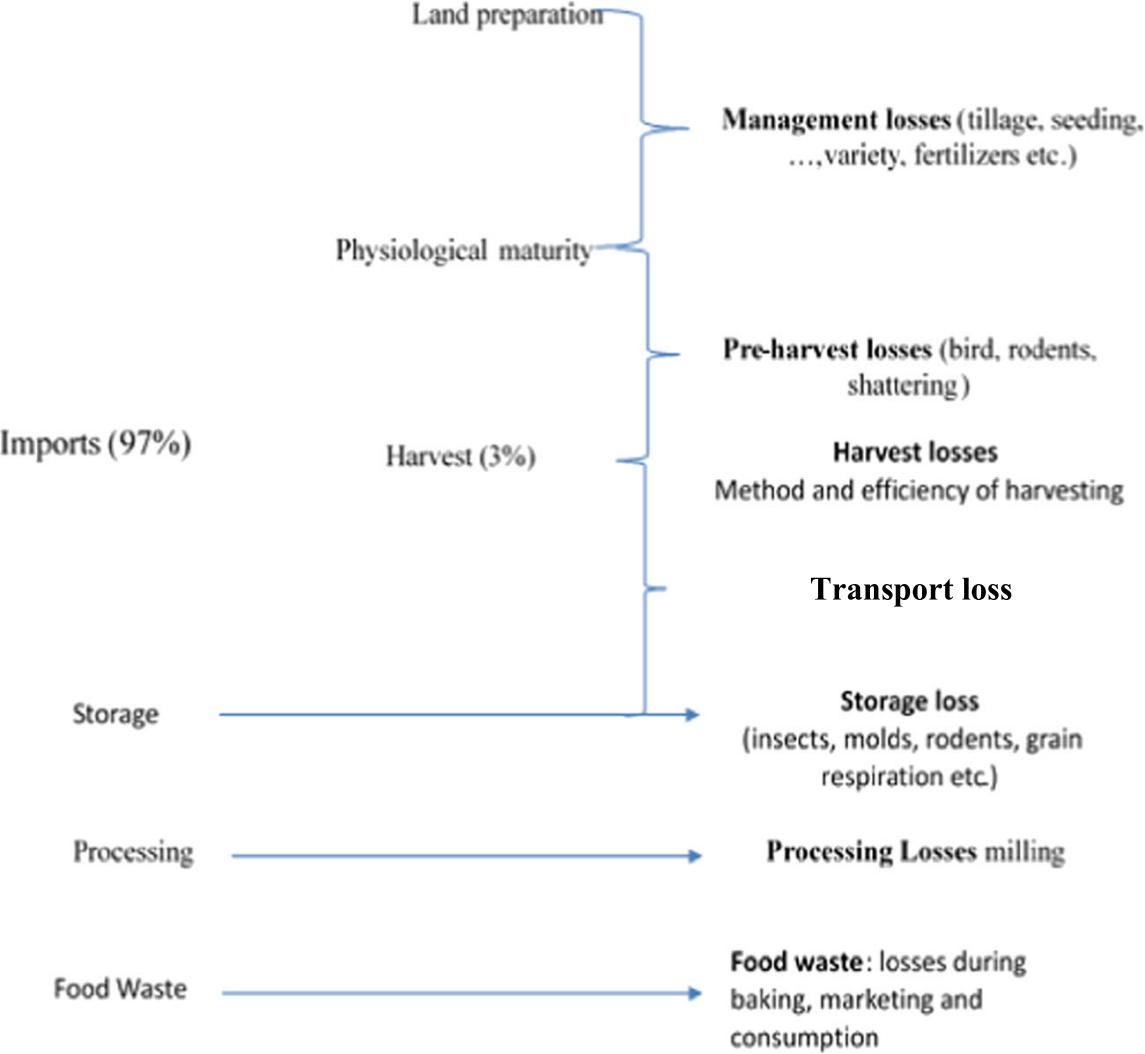
Wheat losses at different nodes of the wheat value chain. Source: depicted by authors

Transport loss mainly occurs due to spillage during loading, movement and unloading. Marketing loss represents food loss mainly at grocery stores and bakeries and include food thrown into the garbage or fed to animals due to overrun of expiry date or loss of freshness of bread. Consumption loss represents food thrown into the garbage or fed to animals due to overrun of expiry date, loss of freshness in bread, and leftovers. The different nodes of the wheat value chain in Jordan and the types of losses that occur at each are presented in Fig. 2.

## Data

In terms of agricultural production, Jordan can be classified into three broad zones namely: the high potential north, the intermediate potential center and the low potential south. Given very limited financial resources for this study, the sample sizes for the estimation of losses and wastage at the different nodes of the value chain were small and hence we are unable to attach any statistical power. However, despite this limitation, efforts were made to get a good representation of the production and consumption patterns in the country by taking representative samples. There could be possible variation in the type and extent of food waste depending on variation factors. Production-related losses may vary by agro-ecology and by size of farms as the production methods, varieties and equipment used, amount of labor deployed, weather, and other factors may be different across different agro-ecologies. There could be differences in the amount of food wasted across household typologies where richer households are expected to waste more as they are likely to buy more but at the same time, the awareness may be higher among some of the richer households and hence may help in reducing waste. There could also be possible differences in efficiency between private and public processing facilities. To avoid possible biases due to such differences, our sample included small-, medium- and large-scale wheat farmers from across all three agro-ecologies (low, medium and high rainfall zones); big and small towns; poor, average and rich households; low-end fast-food restaurants serving mainly the poor, restaurants catering for the middle income portion of the population, and high-end restaurants that target the rich; old, average and new trucks transporting wheat and governmental and private mills.

For pre-harvest and harvest loss measurements, a sample of 30 wheat fields belonging to 30 farmers was selected. The sample was distributed uniformly with 10 fields randomly selected from Irbid in the North, 10 from Mushagar in the Center, and 10 others from Karak in the South.

Measurement of transport loss mainly focused on losses due to spillage during loading and unloading as well as on the road. To this end, a random sample of 30 trucks was selected and tracked from the port storage at Aqaba to the other three storage silos inland.

For storage loss measurement, with permission from the management of the silos, three compartments (called cells) were randomly selected and reserved from the silo complexes in each of the four locations. The sample cells in the three inland silos were left untouched for 4 months (June – September). However, for the Aqaba port silos, the cells were left untouched for only two months as the main use of this silo is to receive and store imported wheat until it is distributed later to the other three inland silos.

While processing losses can occur at the time of converting wheat grain into different products (flour, pasta, and traditional foods such as *bourgol* and *frikeh*), the share of *bourgul*, *frikeh* and pasta in total wheat production in Jordan is very small. Therefore, processing loss measurement in this study focused on flour milling losses. Out of a total of 16 mills, two which included the only government mill and one mill randomly selected from the 15 private mills, were included in the sample and measurements were taken daily for a total of 30 days.

Measurement of losses during marketing were determined by taking random samples of 20 grocery stores and six bakeries and patisseries from the three zones (North, Center and South). For measurement of food waste, a sample of 100 households and 100 restaurants (including 2 university cafeterias) were included in the sample. The sample households and restaurants were uniformly distributed among the three zones (North, Center, and South) of Jordan.

## Materials and methods

In this study we followed the life cycle approach suggested by Bellemare et al. (2017), for measuring food loss and waste along the wheat value chain in Jordan – from farm to fork. This approach was used because there is growing concern that estimates of food loss and wastage are exaggerated as food that has been put under alternative uses is also counted as waste while some of it is indirectly consumed by humans. By using the life cycle approach, we were able to make a distinction between food that is no longer available for direct consumption by human beings and that which is consumed indirectly through consumption of meat from animals and/or birds that are fed with leftover bread.

As there are no credible and consistent protocols for food loss and wastage measurement at each node of the wheat value chain, we also developed measurement and estimation protocols which are consistent with the standards for accounting and reporting food loss and wastage developed by an international initiative led by the World Resources Institute (WRI) (Hanson et al. 2016). For the sake of precision, calculations of losses were made after standardization of all weight measurements throughout the entire value chain into 12% moisture content. In the interest of space, brief and abridged descriptions of the protocols used for measurement or estimation of losses and/or wastage at the different nodes of the value chain are provided below. The longer and more detailed descriptions of the protocols are available upon request.

### Protocol used for measuring pre-harvest loss (PrHL)

For the measurement of pre-harvest loss, the protocol described by Sattar et al. (2015) was used with some modifications. Loss measurements were done at the physiological maturity stage. If the crop growth in the field was visibly homogenous, a random sample of six 1 m × 1 m quadrats (replicates) distributed across the whole field were taken in such a way that all were at least 5 m away from the border of the field. However, if the crop growth in the field was visibly heterogeneous, then the field was classified into poor, medium, or good growth and their respective share (%) in the total area of the field estimated. Then, a random sample of 2 quadrats from each category (making a total sample of six quadrats from the entire field) was taken making sure that each quadrat was 5 m away from the border. Regardless of the homogeneity/heterogeneity of the field, we made sure that the distance between each replicate was at least 20 m.

### Protocol used for harvest loss (HL) measurement

Losses that occurred at the harvest stage are due to the method of harvesting (manual or combine harvester) and due to poor calibration of the combine harvesters. For measuring harvest losses, three options were compared: A) Manual harvesting and hand threshing; B) Mechanical harvesting using farmers’ combines with their own calibration; and C) Mechanical harvesting using farmers’ combines but calibrated by an expert from the research team. Most Jordanian farmers have moved away from manual harvesting into mechanical harvesting. If carefully done, manual harvesting gives maximum yield and minimum harvesting loss (Clay et al. 2012). Therefore, the loss obtained from this measurement (item A above) was used as the benchmark against which all other harvesting and threshing options considered in this study were compared. The differences between items A and B and A and C give estimates of the harvest-related losses endured by farmers.

### The protocol used for measuring management-related losses in the field

Measuring management related losses during crop growth is not straight forward as it represents the yield gap not only arising from the use of obsolete varieties but also the use of suboptimal agronomic practices and inherent inefficiencies of the farmer. This is even more difficult when the intention is to make physical measurements. As a result, crop simulation models are often used to make estimations about yield gaps which essentially represent management/agronomic losses (Lobell 2013). Since no adequate data are available for calibrating a suitable crop simulation model for this task, the Stochastic Frontier Production Function (SFPF) approach (Battesse and Coelli 1995) was used to indirectly measure management related losses. Suppose that the estimate of the average productive efficiency from the econometric estimation of a stochastic frontier production function (SFPF) is given by A (in %). Then, total loss on the field (TFL) is calculated as 100%-A. We then indirectly calculated the total management loss on the field (ML) as: ML = TFL – PrHL – HL where PrHL is pre-harvest loss and HL is harvest loss (both of which are measured using protocols described in Sections 4.1 and 4.2).

### Protocol used for measuring storage losses

Storage losses are best estimated using models such as the PHAST-FEM (Montross et al. 2002), as they use biophysical relationships based on historical ambient and in-bin temperature, relative humidity (RH), pest activity and grain biology. Due to the lack of in-bin temperature and RH measurements in the Jordanian silos, an attempt was made to carry out physical measurements for storage losses using rudimentary methods. Furthermore, due to the limited access to silos, storage loss-related measurements were taken only for four monthly data points covering the period July–October in three out of the four silos while an additional measurement for November was made in one silo. Grain sampled from the silos and kept in hermetic bags were used as the reference weight against which grain samples taken directly from the silos every month were compared. The average difference in the weights between the grain in the hermetic bag and those sampled from the silos each month were considered to provide an estimate of storage loss. As measurements were taken only for four months, some ad-hoc assumptions were made for the estimation of losses during the months for which measurements were not taken.

### Protocol for measuring transport losses

Physical measurements were used to compute transport losses. Transport losses were computed as the difference in the weight of trucks loaded with wheat grain before departure from the silos in the port of Aqaba and after arrival of the same shipment at one of the long-term storage silos inland. As local production represented only 3% of total national wheat supply, the same level of loss as that measured for imported wheat is assumed for local production as well.

### Protocol for measuring processing losses

According to the standard protocol (FMA 2016), four different types of measurements can be used to measure milling loss during a fairly long period of time such as monthly losses. While these calculations provide good insights in terms of what is actually happening, tracking each of the variables for a long period especially in mills where there are huge movements in and out of the mill with large carryovers to the next period (e.g. end of the month) could be particularly difficult. In such situations, another protocol, which we call daily loss, proved to be much easier and hence was used in this study. Daily loss was calculated as the difference between the total weight of wheat grain that is milled in one day and the total weight of the flour produced during that day (both converted to 12% moisture content equivalents).

### Protocol for measuring food waste

Food wastage can be caused by various factors and can take different forms including: 1) loss in quantity of food prepared for consumption, especially when leftover food (left on the plate or even not served) is thrown away; 2) food wasted when it is thrown away after it is prepared due to its deterioration in quality and edibility, making it unfit for human consumption; 3) processed, semi-processed and raw food items thrown away (by households, grocery stores, bakeries, etc.) because they have reached the end of their shelf life (or expiration date).

Food waste at households, restaurants, grocery stores, and bakeries was based on survey data where we calculated the amount of loss as the difference between the amount of flour/bread coming in (purchased) and the amount that was sold/consumed. During the survey, the appropriate individual responsible for preparing food in the household (often a woman), the restaurant or university was interviewed to give estimates of the percentage of food thrown away: a) before food is prepared because of expiration of usage date, b) after it was prepared but before it was served because it was prepared excessively or because it did not come out well (or because it was spoiled due to the elapse of too long a period after preparation) and c) after it was prepared/ordered because it was excessive. For comparison purposes, physical measurement of food waste after consumption at restaurants was carried out (please note that the food waste after consumption is only one aspect as there is also wastage of bread which has not been served which represents order/preparation in excess of demand which we estimated using survey data).

The following formula was used to compute food wastage during or after consumption:

$$ \mathrm{Total}\ \mathrm{amount}\ \mathrm{of}\ \mathrm{food}\ \mathrm{waste}=\sum \limits_i{\mathrm{Waste}\ \mathrm{factor}}_i\ast {\mathrm{Scaling}\ \mathrm{factor}}_i $$

Where:

*i* = category of: households (poor, medium and rich), restaurants (high-end, middle-class and low-end restaurants), grocery stores and bakeries (small, medium and large stores/bakeries);

*Waste factor*_*i*_ represents the average amount of wasted bread by a single household/restaurant, grocery store/ bakery in category *i* in a given period of time;

The *scaling factor*_*i*_ is the total number of households, restaurants, grocery stores, bakeries in category *i* throughout Jordan.

## Results and discussion

### Pre-harvest loss

The average farm size in Jordan is 6 ha. The area-weighted national average pre-harvest losses was about 12.3 kg/ha with the highest loss (14.9 kg/ha) registered for the producers in the southern regions of Jordan. Therefore, every farm is losing (at pre-harvest) an average of 73.8 Kg of wheat. Using the minimum wheat grain price of 370 JD/tonne, the total value of wheat that could be saved per farm was 27.31 JD and using area weights for upward aggregation of regional estimates into national figures, the total loss in the country was estimated at 287.01 ton/ year and 106,193.7 JD (Table 2).

**Table 2:** Estimation of pre-harvest loss at the national level per year

Location/variable	Total wheat Area (ha)	Pre-harvest loss (kg/ha)	Total loss in the country (tons/year)	Minimum value of grain loss
North	12,660.07	11.03		
Central	2992.38	12.61		
South	7365.86	14.88		
National average (area-weighted)		12.47		
Total	23,018.30		287.01	104,465.12

Source: Authors’ calculation

### Harvest loss

Averages yields using manual harvesting in (kg/ha) for north, central and south regions were 3832, 3860, and 2586 respectively (Table 3). The national average yield (using area weights for upward aggregation) was 3437 kg/ha. Yields from mechanical harvesting using farmers’ own calibration for north, central and south were 3578, 3369 and 2385 kg/ha respectively. The national weighted average yield was 3169 kg/ha. The corresponding figures for mechanical harvest using expert calibration for the north, central and south were 3674, 3611 and 2662 kg/ha respectively. The area-weighted average yield was 3342 kg/ha.

**Table 3:** Estimation of yield for manual, mechanical harvest with and without calibration and harvesting by using small combines in Jordan

Location	Manual-Average production (kg/ha)	Mechanical-expert-calibration-Average production (kg/ha)	Mechanical-farmer-calibration-Average production (kg/ha)	Mechanical-small research Combine-Average production (kg/ha)
North	3832.0	3673.9	3578.0	3692.2
Central	3860.0	3610.6	3368.6	3628.6
South	2586.4	2662.4	2385.4	2675.6
National average (simple)	3426.1	3315.6	3110.6	3332.1
National average (weighted)	3437.0	3342.0	3169.1	3358.6
Loss relative to manual harvesting (Kg/ha)	0.00	95.1	267.9	78.5
Loss relative to calibrated combines				
Estimation of Average National loss				
	Loss (kg/ha)	Total loss in the country (tons/year)	Minimum value of grain that can be saved	
Total loss due to mechanical harvesting (farmer calibration)	267.93	6167.25	2,281,882.72	
Total loss due to mechanical harvesting (expert calibration)	95.07	2188.36	809,691.88	
Total loss due to mechanical harvesting (small research combine-harvester)	78.46	1805.94	668,198.58	
Loss from mechanical harvest (due to difference in calibration)	172.86	3978.89	1,472,190.84	
Loss that can easily be avoided by hiring an expert to calibrate farmers’ combine harvesters	172.86	3978.89	1,472,190.84	

Source: Authors’ own calculations

Using the yield from manual harvesting as the reference yield, the average yield losses due to mechanical harvesting using farmer calibration and expert calibration and the small research combiner were 267.9 kg/ha, 95.1 kg/ha and 78.5 kg/ha which represented a total national loss of 6167.25 tons/year, 2188.36 tons/year and 1805.94 tons/year, respectively.

By improving the calibration of their combines alone, farmers could reduce loss of 172.9 kg/ha or nationally 3978.89 tons/year. This loss, which can easily be avoided by hiring an expert to calibrate farmers combine harvesters, was estimated at 64JD/ha. Currently, there is no market for combine harvester calibration in Jordan. However, farmers could make additional profit of at least 34JD/ha if they were to pay even as much as 30JD/ha to hire an expert to calibrate a combine harvester.

### Management losses

The observed (actual) average yield (YO), for the randomly selected samples from the three regions (north, central and south) was 2364.7 kg/ha (Table 4). The efficiency level of the typical farmer was estimated at 80.3%, which shows that on the average farmers are losing 579 kg/ha out of a total potential production at physiological maturity of 2644.9 kg/ha. This shows that the total potential yield in the absence of all preharvest, harvest and postharvest losses was 2943.8 kg/ha. Given that the total loss between physiological maturity and harvest was measured to be 280. 2 kg/ha, the total management-related loss is 298.8 kg/ha (2943.8–280.2). This translates into a total national loss of 6878.71 tons/year (0.76%).

**Table 4:** Estimation of management losses for wheat in Jordan

Mean yield (ton/ha)	2.34
Observed or actual average Yield (YO)=	236.47
Pre-harvest loss (PRHL) in kg/ha=	12.3
Harvest-loss (HL) in kg/ha=	267.9
Total pre-harvest and harvest loss (kg/ha)	280.2
Potential yield at maturity date (kg/ha)	2644.9
Total pre-harvest and harvest loss as % of total potential production of wheat in Jordan (%) =	9.52%
Average efficiency level from SFPR (%) =	80.3%
Potential Yield in the absence of all losses (YP) (kg/ha) =	2943.8
Total production loss (as % of potential yield) =	19.7%
Agronomic loss (kg/ha) =	298.8
Total agronomic loss as % of total potential production of wheat in Jordan (%)	10.15%
Total wheat area in Jordan in ha=	23,018
Total agronomic loss in Jordan (tons per year) =	6878.71
Average annual imports of wheat in Jordan (tons/year)	851,115
Total amount of wheat that is actually available in Jordan (tons/year)	904,980.56
Total amount of wheat that should have been available in Jordan (tons/year)	918,170.34
Total agronomic loss as % of total actual production and imports in Jordan (%)	0.76%

Source: Authors’ own calculation

### Transport losses

The total transport loss was estimated at 0.29% which translated to 2553.38 tons/year. It is important to note here that the quantity lost during transport depended on the kinds of trucks (old vs. new and dumpster vs. semi-trailer trucks) where old dumpster trucks with holes were found to be the ones with the highest loss. Were Jordan to upgrade all the old trucks transporting wheat, it would have prevented the loss of 508 tons of wheat every year.

### Storage losses

After converting all weight measurements into their 12% moisture content equivalents, the loss during the period July–November for which actual measurements were taken was 6.65% (33,269ton/year) with the highest loss 13,702.24 tons (2.74%) occurring in the month of August followed by 10,893.14 tons (2.18%) in September (Tables 5). As measurements for the months between December and June were not taken, ad-hoc assumptions were made to generate estimates of loss during this period. For example, because the temperature in Jordan drops between October and March to levels which are prohibitive of insect and mold growth, we assumed a very low storage loss of 0.05% per month for this season. Whereas, as temperature starts to rise starting from April, higher and exponentially increasing monthly loss rates are expected until August after which temperature starts to fall leading to lower losses as well. Based on experience, the storage loss in June is assumed to be comparable to the measured loss in September (0.29%) while that of May is assumed to be 40% less than that of June and the loss in April is also assumed to be 40% less than that in May. Based on these ad-hoc assumptions, the total storage loss per year was estimated at 11.10% or 55,546.33 tons/year. However, the research team does not have full confidence on the validity of this figure as there are no past data or studies that can be used to substantiate the validity of the assumptions that were made. Therefore, the storage loss of 6.65% that is measured for the period July–November is reported as the minimum estimate of the annual storage loss in Jordan.

**Table 5:** Average values of various parameters used for measuring storage loss based on actual measurements taken from the North, Jwaydeh and Russeifa silos

Average of all silos	July	August	September	October	November	December	January	February	March	April	May	June	Total for the year	%
MOISTURE %	**11.23**	**11.01**	**11.53**	**11.53**	**11.53**									
HERMETIC BAG 50KG	**50.00**	**49.10**	**48.95**	**48.93**	**48.91**									
HERMETIC BAG 50KG (12% MC equivalent)	**50.44**	**49.52**	**49.38**	**49.36**	**49.33**									
Difference in Hermetic bag (at 12% MC)		**0.91**	**0.14**	**0.02**	**0.02**									
% LOSS IN HERMETIC BAGS (AT 12% mc)	**1.08%**	1.81%	0.29%	0.05%	0.05%								**3.27%**	
Total loss in Hermetic bags (12% MC equivalent)) out of the total monthly stored wheat grain of 500,000 tons	**5420.00**	**9033.33**	**1425.76**	**238.31**	**238.42**	**0.00**	**0.00**	**0.00**	**0.00**	**0.00**	**0.00**	**0.00**	**16,355.82**	**3.27%**
SILOS sample of 50 kg volume	50.00	47.61	46.71	46.66	**46.620**									
SILOS sample of 50 kg volume (12% MC equivalent)	50.44	48.14	46.96	46.91	**46.869**									
Difference in SILOS (at 12% MC)		2.29	1.19	0.04	0.04									
% LOSS IN SILOS (at 12% mc)	2.73%	4.55%	2.46%	0.09%	0.09%								**9.92%**	
Total loss in SILOS (12% MC equivalent)) out of the total monthly stored wheat grain of 500,000 tons	**13,641.34**	**22,735.57**	**12,318.90**	**463.89**	**464.32**	**0.00**	**0.00**	**0.00**	**0.00**	**0.00**	**0.00**	**0.00**	**49,624.02**	**9.92%**
Difference between Hermetic bags and Silos in sample of 50 Kg (12% MC)	**1.64%**	**2.74%**	**2.18%**	**0.05%**	**0.05%**								**33,268.20**	**6.65%**

Source: Authors’ own calculation

**Table 6:** Summary of milling loss based on 30-days data from Jwaydeh Mills

	Bran for animals	Bran for humans				
Suppose that:			Tons per month	Total %	Animals	Humans
a = Raw/dirty wheat used in first break			5236.0			
b = flour produced			4519.5			
c = screenings/dockage			0			
d = mill-feed/offal (waste material)			0			
e = bran	676.42	16.7	693.1	13.24%	12.92%	0.32%
Calculation methods						
1. Raw/dirty wheat				13.68%		
2. First break				13.68%		
3. Total products				13.30%		
4. Mill gain (−) or loss (+) excluding bran for animal and human consumption			In tones	23.34		
			In %	0.45%		

Source: Author’s own calculation

### Processing losses

Data obtained from the sample private mill was not reliable. Therefore, the results were not included in the analysis and the results from the public mills were used to estimate total national milling loss (Table 6). The sum of the daily amounts of wheat grain that entered the Jwaydeh public mill for the month of October 2016 was 5235.94 tons. 4519.47 tons of different kinds of flour were produced which included 13.24% of bran, out of which 12.92% was used as animal feed and 0.32% for human consumption (mixed with bleached flour to make whole wheat flour). There was also milling loss of 0.45%. The total processing loss was therefore 13.68% (which includes bran fed to animals and milling loss).

### Food waste

Losses at bakeries and patisseries were estimated at 13565.25tons/year (1.55%). The total loss in grocery stores was estimated at 770.02tons/year out of which the majority (720.25 ton/year) was in the form of bread that was thrown into the garbage while the remaining (49.77 ton/year) is flour disposed due to expiration.

Based on the physical measurements carried, the total amount of bread lost at restaurants was estimated at about 26,392tons/year (23% of total bread served in restaurants). This is in quite a contrast with the estimate (4.52%) based on interviews with restaurant managers showing that reported wastage is often underestimated which should not come as a surprise given the cultural and religious taboos of throwing food out. In addition, based on the survey data collected from the 100 sample households, the total wheat-based food disposed during consumption in households was estimated at 26.86% of total food purchased by the typical household, out of which 5.36% is estimated to have been given to other people for consumption, 18.12% fed to animals, and only the remaining 3.38% thrown into the garbage (Table 7). This implies that the total amount of wheat that was processed but became unavailable for direct human consumption was 21.51% (18.21% + 3.38%) of total national wheat supply (114,411 tons/year).

**Table 7:** Food waste during consumption by households

Item	Food wasted after serving (kg/day) (at 12% MC Wheat Equivalents)			Food wasted untouched (kg/day) (at 12% MC Wheat Equivalents)			Total wastage in the total Jordanian households (at 12% MC Wheat Equivalents)			
	Garbage	Human	Animal	Garbage	Human	Animal	Garbage	Human	Animal	Grand total
White bread (khubz)	0.00342	0.01499	0.00085	0.00062	0.00124	0.00808	7986.33	32,098.91	17,662.08	57,747.33
Medium white wheat bread (khubz)	0.00039	0.01165	0.00124	0.00093	0.00000	0.00031	2610.92	23,037.50	3071.67	28,720.08
Black (whole) wheat bread (khubz)	0.00404	0.00039	0.00450	0.00000	0.00062	0.00132	7986.33	1996.58	11,518.75	21,501.66
Other white bread (baguette, slices, buns, rolls, etc.)	0.00000	0.00000	0.00047	0.00000	0.00777	0.01553	–	15,358.33	31,638.16	46,996.49
Other whole wheat bread (baguette, slices, buns, rolls, etc.)	0.00000	0.00155	0.00000	0.00000	0.00000	0.00000	–	3071.67	–	3071.67
Pasta (macaroni, spaghetti, tagliatelle, lasagna, etc.)	0.00440	0.00000	0.00070	0.00704	0.00000	0.00440	22,611.30	–	10,088.12	32,699.42
Bourghul	0.00020	0.00049	0.00489	0.00000	0.00000	0.00000	386.52	966.30	9662.95	11,015.76
Friekhe	0.00000	0.00000	0.00000	0.00000	0.00000	0.00000	–	–	–	–
Cakes	0.00000	0.00000	0.00000	0.00000	0.00000	0.00000	–	–	–	–
Cookies and biscuits	0.00000	0.00000	0.06157	0.00000	0.00000	0.00000	–	–	121,753.17	121,753.17
Manaeish	0.00000	0.00000	0.00000	0.00000	0.00000	0.00000	–	–	–	–
Flour	0.00391	0.00078	0.02003	0.00000	0.00000	0.00968	7730.36	1546.07	58,750.74	68,027.17
	0.01635	0.02985	0.09426	0.00859	0.00963	0.03931	49,311.76	78,075.36	264,145.64	391,532.76
Total amount of wheat-based products purchased by the households (tons/year)										531,999.06
Total number of households in Jordan										1,977,534.00
Total national amount of food wasted at household level (tons/day)							49.31	78.0	264.15	391.53
Total national amount of food wasted at household level (tons/year)							17,998.79	28,497.51	96,413.16	142,909.46
Total loss (%)							3.38%	5.36%	18.12%	26.86%

Source: Authors’ own calculation

### Discussion

Table 8 provides the detailed accounting of losses from the field (for local production) and the port (for imports) all the way to the fork. Grain was tracked along each node in the entire value chain where cumulative losses that were incurred in preceding nodes were deducted from the total amount entering the subsequent nodes. To make the estimation consistent, all wheat and wheat products at each node were converted into uniform moisture content of 12%. Accordingly, total management (agronomic) losses were estimated at 10.15% of potential production while pre-harvest and harvest losses were 0.42% and 9.10% respectively. However, given that local production is only 3% of total food supply in the country, the total loss in the field (including all agronomic, pre-harvest and harvest losses) was estimated at only 1.47% of total food supply in the country. Measurement for transport loss was carried out only for wheat grain transported from the port to the different silos. It is assumed that it will be the same also for loss during transport from the field (for local production) to the silos. Loss during transport from ports and fields to the silos was only 0.29%.

**Table 8:** Summary for all the losses occurring at different nodes of the wheat value chain in Jordan

Item	Agronomic loss	Pre-harvest loss	Harvest loss	Port	Local production	Silos	Mills	Bakeries	Stores (flours)	Stores (bread)	Households	Restaurants
Quantity entering (ton/ year)				851,115.00	23,420.00	871,998.85	814,010.93	632,388.81	70,265.42	61,898.22	525,778.93	92,491.41
Storage loss				0%	0.00	0.07	0.00	0.00	0.00	0.00	0.00	0.00
Total storage loss (tons/year)				0.00	0.00	57,987.92	0.00	0.00	0.00	0.00	0.00	0.00
Loss during transport to silos (%)				0.29%	0.29%	0.00%	0.00%	0.00%	0.00%	0.00%	0.00%	0.00%
Loss during transport to silos (tons/year)				2468.23	67.92	0.00	0.00	0.00	0.00	0.00	0.00	0.00
Total processing loss (%)				0.00%	0.00%	0.00%	13.68%	0.00%	0.00%	0.00%	0.00%	0.00%
Total processing loss (tons)				0.00	0.00	0.00	111,356.69	0.00	0.00	0.00	0.00	0.00
Disposed wheat-based products (%)								2.12%	0.07%	1.15%	21.51%	3.02%
Disposed wheat-based products (tons)								13,406.64	49.19	711.83	113,095.05	2793.24
Total loss (tons/year)	**6878.71**	**282.34**	**6167.00**	**2536.15**		**57,987.92**	**111,356.69**	**13,406.64**	**49.19**	**711.83**	**113,095.05**	**2793.24**
Loss as % of (imports + local production)				0.00		0.07	0.13	0.02	0.00	0.00	0.07	0.00
Cumulative loss staring from port or farm (tons)				2536.15		60,524.07	171,880.77	185,287.41	185,336.60	186,048.43	299,143.47	301,936.71
Cumulative loss staring from port or farm (%)				0.29%		6.92%	19.65%	21.19%	21.19%	21.27%	34.21%	34.53%
Loss at each node as % total local actual production (%)	10.15%	0.42%	9.10%	4.71%		107.65%	206.73%	24.89%	0.09%	1.32%	209.96%	5.19%
Loss at each node (as % of imports + local actual production)	**0.76%**	**0.03%**	**0.68%**	**0.29%**		**6.63%**	**12.73%**	**1.53%**	**0.01%**	**0.08%**	**12.93%**	**0.32%**
Rank of loss (considering both local production and imports)				**6**		**3**	**2**	**4**	**8**	**7**	**1**	**5**

The total annual storage loss was estimated at 11.1%. However, as no physical measurement was taken for the period December – June, we had to make ad-hoc assumptions with which we are not comfortable. Therefore, we consider here only the storage loss for the period July–November for which physical measurement was taken. During this period alone, storage loss in the country was estimated at 6.65%. Likewise, the total processing loss (loss at flour mills) was estimated at 12.75% of total wheat supply in the country. Over 97% of the total loss at the flour mills represents bran that is sold to farmers as animal feed. The loss at bakeries, grocery stores and restaurants were, respectively, 1.55%, 0.09% and 0.32%. With a total loss of 12.95%, food loss and wastage at household level ranks first followed by that which is lost during milling (12.73%). However, given that almost all the loss at the mills is given to animals, one can argue that it is not lost.

In summary, the total amount of lost wheat is estimated at 301.9 thousand tonnes (34.53%) which, using a five-year average price of US$350 /ton, is equivalent to US$105.3 million per year. Food lost or wasted also means energy and water lost/wasted (Verma 2015; Cuéllar and Webber 2010). The average amount of water needed to produce one kilogram of wheat is 1.1M^3^ (Yigezu et al. 2014 for Syria; Hussain et al., 2003 for Pakistan). The average amount of water needed to process 1 kg of wheat equivalent flour into bread is 0.78 l/kg (Food, 2009). Likewise, the average energy needed for producing 1 ton of wheat was about 3223 MJ (Fox and Fimeche 2013) and for baking is about 5338 MJ per ton (Beech and Crafts -Lighty 1980). Using the above parameters from previous studies, the total amount of water lost due to total food loss and wastage (including the food given to animals) in Jordan is estimated at 348 million m^3^ (99% for production and only 1% for processing) valued at $41 million. The wheat loss in Jordan also implied a total loss of 3.68 million GJ of energy (27.64% for production and 73.36% for processing) which is equivalent to 115 million liters of diesel with an estimated value of $70 million.

Households, restaurants, bakeries and grocery stores were asked what they do with unconsumed or unsold bread and wheat-based products (particularly flour). Almost all of them said they give it to animals. However, due to religious and cultural values in the region, the estimates of bread used as animal feed are likely over estimated. Stacks of bread thrown inside or beside garbage dumpsters are a common scene in major cities in Jordan. Interviews with 10 municipality workers who are responsible for lifting garbage dumpsters revealed that bread constitutes between 10% and 30% of the total amount of garbage collected. Therefore, the estimates of bread used as animal feed is likely to be overestimated while that which is thrown into the garbage is under estimated. But even if the respondents’ figures were accurate, the total amount of food that is thrown away into the garbage was 84,385 tons/year (9.65%). Moreover, feeding animals with bread, the production of which requires substantial amounts of energy, water and labor, is a waste of natural and financial resources. Using the import prices of wheat ($350/ton) and barley ($250/ton) and the estimated cost of producing and processing 1 kg of wheat into bread, Jordan is losing 43% and 48% respectively of total protein and energy for every 1US$ spent on bread that is fed to animals instead of equivalent value of barley. Alternatively, if Jordan were to feed the animals with raw wheat instead of bread, the country would have saved 31% of both energy and protein for every dollar spent on bread fed to animals.

## Conclusions and recommendations

Being a major global issue, food waste and loss remain relatively unexplored topics especially in the Middle East in general and in Jordan in particular. While there are rough estimates of the total global food losses, most of them are either based on expert opinions and hence vary widely or they do not provide clear explanation on what was being estimated, how the estimation was made, and when. More importantly, only few of them provide detailed breakdown in terms of where in the value chains exactly these losses are taking place. Food losses and waste can happen at different nodes of the value chain, namely: in the field during the growing periods of the crop, at harvest, at storage and during transport, processing, marketing, and consumption. Having estimates of losses at each of these nodes will be highly valuable for policy making and targeting and prioritizing interventions.

In this paper, we attempted to estimate the total wheat loss that occurs at each node across the entire wheat value chain in Jordan from the farm (for local production) and port (for imports) to the fork. Except for the pre-harvest, transport and marketing nodes, substantial losses are recorded at each of the other nodes along the wheat value chain. Using a combination of physical measurements and surveys, we estimated that at least 34% of total wheat supply in Jordan (both from local production and imports) is lost or wasted – costing the country about US$105 million per year directly.

This research adds value to the global community in general and Jordan in particular in many different ways. 1) It contributes to the literature both in terms of providing a complete set of protocols for food loss and wastage measurement along the wheat value chain (from farm to fork); 2) it also contributes to the literature by providing estimates of food losses and waste at each node of the value chain using a combination of physical measurements and scientifically defendable estimation procedures which are then aggregated into national levels – an attempt, which to the best of our knowledge, has not been made by any other study. 3) In the course of this study, important discussions have already started to emerge, and the findings have provided useful information which we believe has contributed to the recent changes introduced by the Jordanian government. It is also expected to continue benefiting the country and other countries in the region with similar production, socio-cultural and political environments.

Postharvest losses were found to be important in Jordan. Particularly, the major losses that occur at the household level where processed food (mostly bread) is either thrown away into the garbage or given away for use as animal feed. Food loss and wastage at household level represent about 12.95% of the total wheat available in the country. The total amounts of water and energy that were expended to produce wheat lost or wasted in Jordan are respectively estimated at 348 million m^3^ and 3.68 million GJ (which is equivalent to 115 million liters of diesel) valued at about $41 million and $70 million respectively.

During personal interviews with heads of the 100 sample households and managers of the sample restaurants, bakeries and grocery stores, it was emphasized that most (84%) of the unconsumed bread was given to farmers for use as animal feed. However, due to religious and cultural taboos in the region, the estimates of bread thrown away are believed to have been underestimated. Some justifications in favor of our argument include stacks of bread thrown inside or beside garbage dumpsters are a common scene in major cities of Jordan. However, even if the respondents were accurately reporting, feeding animals with bread which has been produced using substantial amounts of energy, water and labor is a waste of natural and financial resources. Using standard conversion factors, we estimated that Jordan is losing 43% and 48% respectively of total protein and energy for every 1US$ spent on bread that is fed to animals instead of the equivalent amount of barley. Alternatively, if Jordan were to feed the animals with raw wheat instead of bread, the country would have saved 31% of both energy and protein for every dollar spent on bread fed to animals.

The findings of this study show that postharvest losses, particularly processed food wastes that are used as animal feed in Jordan are very high. Disregarding the nutritional advantages of feeding raw wheat, given the high cost of energy, labor, water and other resources for processing, using bread as animal feed is not an optimal option for Jordan. These phenomena signal an urgent need for individuals, civic society organizations, NGOs and the government to make concerted efforts towards awareness raising and measures targeting reduction in losses, particularly food waste at household levels that end up as animal feed.

Jordan cannot afford to and should not continue with such a great magnitude of loss. Given the high levels of water and energy constraints in the country and the very high opportunity cost of the financial resources devoted to the production and import of wheat in the country, investment towards reducing food loss and wastage is imperative. While finding solutions to the various kinds of losses will require more research, some minor behavioral, cultural and institutional changes as well as policy changes at individual, society and national levels could go a long way in reducing these losses. Among other matters, the following changes could help to reduce food loss and wastage in Jordan:

1. Awareness should be created among society about the magnitude of wheat loss and wastage and its moral implications and resource use inefficiency.
2. Options for smaller package sizes or bundles of 0.1 kg, 0.25 kg, 0.5 kg and 1 kg and even piece-meal sales of bread should be provided by bakeries. These will enable smaller and prudent families to buy only the quantity of bread that they consume.
3. Jordan should study carefully the option of replacing flour subsidies with bread subsidies. Furthermore, introducing a voucher system which enables the subsidy in the country to target only poorer families and motivate them to be careful in their use of bread would be more effective in both reducing bread waste and also improving nutritional diversity of the poor. Studying better approaches (including the recent approach adopted by the Egyptian government) of providing vouchers to the needy while bread is sold at its actual production cost might be beneficial.
4. Increased investment in research to reduce losses at each node of wheat value chain, particularly losses occurring during consumption at the household level.
5. Installation of equipment for the measurements of temperature and relative humidity in all Jordanian silos to help monitor the grain in the silos as well as for generating better and more reliable estimates of storage losses.

Over the course of the production of the dissertation which led to this paper, there have been many discussions on the topic of food loss and wastage in Jordan (see for example Duwayri 2016). In January 2018, the Jordanian government reduced the amount of subsidy. Instead, poor households were given a certain amount of cash every year as compensation for the higher bread prices. While this is a move in the right direction, whether it is effective in addressing the problem and if its implementation can be improved needs to be studied.

One of the major limitations in this study is that due to limited funding, the sample sizes were small. Larger sample sizes would provide statistical confidence and this will be a matter for future endeavors.

## Footnotes

1 As of January 2018, the prices of *mowahad* and *mohassan Khubz* have been increased from 0.16 to 0.32JD/kg and from 0.25 to 0.50 JD/kg respectively.
